# Supplementary material for: Causal Relationship between Aging and Anorexia Nervosa: A White-Matter-Microstructure-Mediated Mendelian Randomization Analysis
Source: Biomedicines. 2024 Aug 16;12(8):1874. doi: 10.3390/biomedicines12081874 (PMC11351342; doi:10.3390/biomedicines12081874)
Supplement: Supplementary file 1 [file biomedicines-12-01874-s001.zip › Supplementary Figure/Figure S4/Singe SNP plot of MR analysis for TL to ubm-b-1805.pdf]

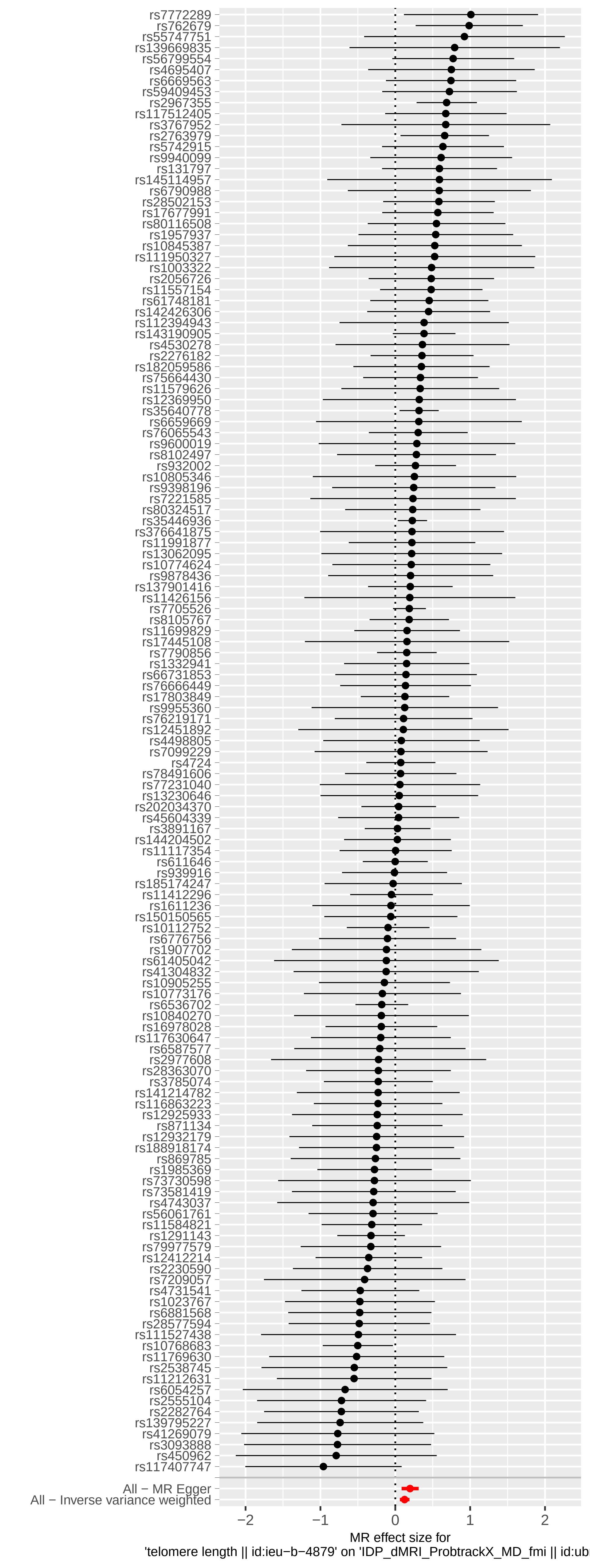
rs7772289
rs762679
rs55747751
rs139669835
rs56799554
rs4695407
rs6669563
rs59409453
rs2967355
rs117512405
rs3767952
rs2763979
rs5742915
rs9940099
rs131797
rs145114957
rs6790988
rs28502153
rs17677991
rs80116508
rs1957937
rs10845387
rs111950327
rs1003322
rs2056726
rs11557154
rs61748181
rs142426306
rs112394943
rs143190905
rs4530278
rs2276182
rs182059586
rs75664430
rs11579626
rs12369950
rs35640778
rs6659669
rs76065543
rs9600019
rs8102497
rs932002
rs10805346
rs9398196
rs7221585
rs80324517
rs35446936
rs376641875
rs11991877
rs13062095
rs10774624
rs9878436
rs137901416
rs11426156
rs7705526
rs8105767
rs11699829
rs17445108
rs7790856
rs1332941
rs66731853
rs76666449
rs17803849
rs9955360
rs76219171
rs12451892
rs4498805
rs7099229
rs4724
rs78491606
rs77231040
rs13230646
rs202034370
rs45604339
rs3891167
rs144204502
rs11117354
rs611646
rs939916
rs185174247
rs11412296
rs1611236
rs150150565
rs10112752
rs6776756
rs1907702
rs61405042
rs41304832
rs10905255
rs10773176
rs6536702
rs10840270
rs16978028
rs117630647
rs6587577
rs2977608
rs28363070
rs3785074
rs141214782
rs116863223
rs12925933
rs871134
rs12932179
rs188918174
rs869785
rs1985369
rs73730598
rs73581419
rs4743037
rs56061761
rs11584821
rs1291143
rs79977579
rs12412214
rs2230590
rs7209057
rs4731541
rs1023767
rs6881568
rs28577594
rs111527438
rs10768683
rs11769630
rs2538745
rs11212631
rs6054257
rs2555104
rs2282764
rs139795227
rs41269079
rs3093888
rs450962
rs117407747
All − MR Egger
All − Inverse variance weighted
−2
−1
0
1
2
MR effect size for
'telomere length || id:ieu−b−4879' on 'IDP_dMRI_ProbtrackX_MD_fmi || id:ub
